# Development of Targeted Drug Delivery System for the Treatment of SARS-CoV-2 Using Aptamer-Conjugated Gold Nanoparticles

**DOI:** 10.3390/pharmaceutics16101288

**Published:** 2024-09-30

**Authors:** Junghun Park, Hyogu Han, Jun Ki Ahn

**Affiliations:** 1 Department of Biologics, Gachon University, Incheon 21936, Republic of Korea; jhpk@gachon.ac.kr; 2 User Convenience Technology R&D Department, Korea Institute of Industrial Technology (KITECH), Ansan 15588, Republic of Korea; ninehyo@kitech.re.kr; 3 Department of Chemistry, Gangneung-Wonju National University, Gangneung 25457, Republic of Korea

**Keywords:** hyaluronic acid, gold nanoparticle, drug delivery, controlled release, one-pot synthesis, poorly water-soluble drug, niclosamide

## Abstract

**Background:** The SARS-CoV-2 pandemic has highlighted niclosamide (NIC) as a promising treatment for COVID-19. However, its clinical application is limited due to its poor water solubility, resulting in low bioavailability. **Methods:** To address this issue, we developed a AuNP-HA-NIC system, which combines gold nanoparticles with hyaluronic acid to enhance drug delivery. Our comprehensive characterization of the system revealed that hyaluronic acid with specific molecular weights, particularly those exposed to electron-beam irradiation between 2 and 20 kGy, produced the most stable nanoparticles for efficient drug loading and delivery. **Results:** Additionally, the AuNP-HA-NIC system exhibits a significant sensitivity to pH changes, which is a critical feature for targeted drug release. Under acidic conditions mimicking the stomach and small intestine, minimal drug release was observed, indicating the effective prevention of premature drug release in the gastrointestinal tract. Furthermore, the integration of a targeting aptamer established specific binding abilities towards the SARS-CoV-2 spike protein, distinguishing it from other coronaviruses. **Conclusions:** As research progresses, and with further in vivo testing and optimization, the AuNP-HA-NIC–aptamer system holds great promise as a game-changer in the field of antiviral therapeutics, particularly in the battle against COVID-19.

## 1. Introduction

COVID-19, declared a pandemic in 2020, has affected more than 700 million people and caused over 7 million deaths by 2024 [1,2]. Despite the availability of SARS-CoV-2 vaccines, new variants of the virus continue to emerge, emphasizing the need for additional treatments. Numerous companies including Merck & Co., Pfizer, and Shionogi have developed anti-SARS-CoV-2 drugs like Lagevrio (Molnupiravir) [3,4], Paxlovid (Ritonavir-boosted Nirmatrelvir) [5], and Xocova (Ensitrelvir) [6], kickstarting a competition for an effective and user-friendly COVID-19 treatment. Among them, Remdesivir, an injectable formulation for COVID-19 treatment [7,8,9], is the most notable and initially showed promising results. However, side effects such as bradycardia and hypotension, which can weaken heart function, have been reported, albeit very rarely.

It was hypothesized that Remdesivir might bind to receptors abundantly present on the surface of cardiac cells, potentially leading to these side effects. After testing its interaction with approximately 350 different receptors, it was confirmed that Remdesivir binds and activates the urotensin receptor, which influences cardiovascular contraction [10]. Therefore, developing new Remdesivir formulations is critical to providing a safer and more effective COVID-19 treatment. These side effects can limit the patient population that can be treated, and different strains of the virus can reduce the effectiveness of treatment, making it necessary to develop different treatment strategies.

Niclosamide (NIC) has been the subject of active research in the fields of anti-cancer [11,12], anti-inflammatory [13], and antiviral treatments [14]. It has proven particularly effective in combating viruses such as Middle East respiratory syndrome coronavirus (MERS-CoV) [15] and severe acute respiratory syndrome coronavirus (SARS-CoV) [16], the precursors to SARS-CoV-2. While the broad-spectrum antiviral ability of NIC was discovered in 2004 [17], the mechanisms behind its effectiveness were only uncovered in 2019. Against SARS-CoV-2, NIC acts by (1) inhibiting the process of endocytosis, (2) reducing autophagy, and (3) halting viral replication [18]. Notably, considerable antiviral activity was demonstrated in SARS-CoV-2-infected VERO cells at a dose of 0.16 μM (57.14 ng/mL) [19]. In this paper, NIC showed 40-fold higher SARS-CoV-2 antiviral activity than Remdesivir and 26-fold higher than chloroquine [20]. These findings highlight the potential of NIC as a potent antiviral agent and underscore the urgent need for further research and development of NIC-based formulations to effectively combat COVID-19.

While the antiviral efficacy of NIC has been demonstrated in many aspects, its low bioavailability has limited its active use in viral treatment. The low bioavailability of NIC is primarily due to its low solubility in water, which makes it difficult for NIC to be absorbed in the body [21]. To maximize therapeutic benefits and reduce potential side effects, various strategies have been employed utilizing water-soluble polymers [22], lipids, surfactants [23], and inorganic materials [24,25]. However, these strategies often face limitations such as complex manufacturing processes, potential toxicity, and inconsistent drug release profiles. To overcome these challenges, various methods, such as utilizing 4-toluic acid dithiobenzoate (TAD) and chemically conjugating with polyethylene glycol (PEG), have been explored to encapsulate NIC in a polymer matrix to improve its release control and bioavailability [21]. While these methods show promise, they also present challenges related to stability and scalability. As an alternative, hyaluronic acid (HA) offers significant potential for NIC formulation. HA is a bio-derived polymer that exhibits different physical properties, such as viscosity, depending on its molecular weight, which has a significant impact on nanoparticle synthesis [26].

In this study, we utilized a one-pot synthesis strategy to load NIC onto AuNP-HA, enhancing bioavailability and achieving controlled release, based on the findings from a previous study. Various molecular weights of HA were obtained through electron-beam irradiation, with smaller polymer sizes observed at higher energy levels. An aptamer targeting the SARS-CoV-2 spike protein was attached to the AuNPs, creating a targeted drug delivery system. The formulated AuNP-HA-NIC showed high drug encapsulation efficiency (DEE) and drug loading capacity (DLC), and its precise targeting was validated using the fluorescent SARS-CoV-2 spike protein, indicating a novel approach in NIC delivery.

## 2. Materials and Methods

### 2.1. Materials

Gold(III) chloride trihydrate (HAuCl_4_, 99.9%), sodium borohydride (NaBH_4_), NIC, phosphate-buffered saline (PBS, pH 7.4), pepsin, hydrochloric acid (1 M), sodium bicarbonate, bovine serum albumin (BSA), tween 20, and NTA-Atto 550 were purchased from Sigma-Aldrich (St. Louis, MO, USA). Ethanol was obtained from Daejung Chemicals and Metals (Siheung-si, Republic of Korea). The medical-grade HA powder was sourced from Hyundai Bioland, with the specified Lot No. HA022002 (Cheongju-si, Republic of Korea). The C2C12 cell line (CRL-1772) and fetal bovine serum (FBS, Cat. No. 30-2020) were obtained from American Type Culture Collection (ATCC, Manassas, VA, USA). Dulbecco’s Modified Eagle’s Medium (DMEM, Cat. No. SH30243) and penicillin/streptomycin (Cat. No. SV30010) were purchased from Hyclone (Logan, UT, USA). Dojindo Laboratories in Japan provided the Cell Counting Kit-8 (CCK-8). Aptamer was synthesized by Bioneer (Daejeon, Republic of Korea), and its sequence is described in Table S1. Spike proteins associated with SARS-CoV-2 and human coronavirus were obtained from Sino Biological (Beijing, China).

### 2.2. Electron-Beam Irradiation of HA

HA powder was dissolved in distilled water (DW). Subsequently, 22.5 mL of the HA solution with a concentration of 10 mg/mL was transferred to a 50 mL centrifuge tube. For electron-beam (e-beam) irradiation, a high-frequency linear electron accelerator was utilized, having an electron energy parameter of 5 MeV and a 50 kW power specification. By adjusting the electric current and modulating the conveyor speed, the e-beam irradiation dose was applied from 1 kGy to 50 kGy. Following irradiation, a freeze-drying procedure, using the Advantage Plus EL-85 model from SP Scientific (Warminster, PA, USA), was applied to the HA samples, ensuring they were preserved for prolonged periods. The polymer dimensions and accurate mass of the treated HA samples were evaluated using a combination of high-performance liquid chromatography (HPLC, 1260, Agilent Technologies, Santa Clara, CA, USA) and a multi-angle light scattering detector (MALS; Dawn Heleos II and Optilab T-rEX, Wyatt Technology Corp., Santa Barbara, CA, USA) as described in a previous study [26]. The dynamic viscosity of the HA samples, after diluting them to 5 mg/mL, was gauged at ambient temperature using a rotary viscometer, model WVS-2M, produced by Daihan (Busan, Republic of Korea).

### 2.3. Synthesis of Niclosamide-Incorporated HA–Gold Nanoparticles (AuNP-HA-NIC)

In 0.8 mL of distilled water (DW), 50 mg of HAuCl_4_ and 8 mg of the e-beam irradiated HA were mixed for 5 min at room temperature. Following the mixing, 19 mL of a NIC solution (8 mg/mL in ethanol) was added to the mixture. Immediately thereafter, 0.2 mL of NaBH_4_ (50 mg/mL in DW) was added. The mixture was stirred continuously for 24 h until the color of the solution transitioned from yellow to red-purple. Subsequently, the precipitate was collected via centrifugation at 17,000× *g* for 15 min and thoroughly washed three times with ethanol.

### 2.4. Characterization of AuNP-HA-NIC

The particle size and morphology analysis of the AuNP-HA-NIC was conducted using a Cs-corrected scanning transmission electron microscope (TEM; JEM-ARM 200F, JEOL, Tokyo, Japan). Absorption spectra were acquired using a UV–visible spectrometer (UV–Vis; Biomate 3S, Thermo Fisher Scientific, Waltham, MA, USA). Colloidal properties including zeta potential and hydrodynamic size were assessed using dynamic light scattering (DLS; ELSZ-1000, Otsuka Electronics Co. Ltd., Osaka, Japan), with samples being adequately dispersed and diluted in DW.

### 2.5. Evaluation of Drug Encapsulation Efficiency (DEE) and Drug Loading Capacity (DLC)

The nanoparticle suspensions were centrifuged at 17,000× *g* for 5 min, after which the supernatants were collected. Drug encapsulation efficiency (DEE) and drug loading capacity (DLC) were assessed by measuring the absorption intensity of the supernatants using a microplate reader (Synergy H1; BioTek, Winooski, VT, USA) within the wavelength range of 300–700 nm. The DEE and DLC were computed according to the equations below [27]:

(1) $$DEE(\%)=\frac{{AMT}_{total}-{AMT}_{free}}{{AMT}_{total}}\times 100$$

(2) $$DLC(\%)=\frac{{AMT}_{total}-{AMT}_{free}}{W_{n}}\times 100$$

In these equations, *AMT_total_* represents the total amount of NIC, *AMT_free_* denotes the amount of free NIC in the supernatant, and *W_n_* is the weight of the nanoparticles after freeze drying. All measurements were conducted in triplicate, with the reported values representing mean values along with standard deviations.

### 2.6. NIC Release Study

The NIC release patterns over time were studied using various solutions containing 0.5% (*wt*/*wt*) tween 20 [28,29,30]: phosphate-buffered saline (PBS, pH 7.4), simulated gastric fluid, simulated intestinal fluid, and simulated blood plasma. The simulated gastric and intestinal fluids were designed to replicate the typical oral administration environment. To make the simulated gastric fluid, 2.0 g of sodium chloride and 3.2 g of pepsin were mixed in DW. The mixture was then titrated with 1 M hydrochloric acid until it reached a pH of 1.5, and the total volume was adjusted to 1 L [26,27]. The simulated intestinal fluid was prepared by dissolving 2.0 g of sodium chloride and 3.2 g of pepsin in DW, keeping a composition akin to the gastric fluid. The pH was then adjusted to 6.8 by adding a measured amount of 1.12 M sodium bicarbonate [21]. For the simulated blood plasma, 50 g of albumin was introduced to 1 L of PBS (devoid of Ca^2+^ and Mg^2+^). For each assay, 10 mg of nanoparticles was dispersed into 50 mL of the respective solution and kept at 36.5 °C with gentle shaking in a shaker incubator. After extracting 1 mL samples at specific time points, they were centrifuged at 17,000× *g* for 5 min and then passed through a syringe filter. The released NIC in these samples was quantified using a microplate reader, measuring the absorbance at 330 nm wavelength. Every experiment was conducted three times to ensure consistency.

### 2.7. Synthesis of Aptamer-Conjugated AuNP-HA-NIC

For the conjugation of aptamers, thiol-functional-group-modified aptamers (50 pM, 0.16 mL) were incrementally added to AuNPs (4 pM, 74.4 μg/mL, 1 mL) in the presence of TCEP (50 pM, 0.32 mL) in 3.52 mL of PBS, totaling a volume of 5 mL, with the comprehensive reagent ratio being 1:2:2 for AuNP–aptamer–TCEP across a span of 24 h. Post-conjugation, the unattached aptamers were eliminated by centrifugation at 15,000 rpm for 10 min.

### 2.8. Targeting Efficiency of Aptamer-Conjugated AuNP-HA-NIC

To investigate the targeting efficiency of the aptamer-conjugated AuNP-HA-NIC, NTA-Atto 550 was attached to the prepared SARS-CoV-2 His-tag spike protein. A total of 200 pmol of spike protein and 800 pmol of NTA-Atto 550 were used, resulting in a final concentration of 1 mg/mL with PBS as the solvent. The mixture of spike protein and NTA-Atto 550 was stirred in the dark for 1 h, with NTA binding to the His-tag portion of the spike protein. The corresponding fluorescence was observed at an excitation wavelength of 550 nm and an emission wavelength of 580 nm. Subsequently, the prepared SARS-CoV-2-NTA-Atto 550 and AuNP-HA-NIC-Apt were reacted in the dark for 1 h at varying ratios of 2:1, 1:1, 1:2, and 1:5, respectively. AuNP-HA-NIC without the aptamer was utilized as a control. Additionally, to verify specificity, the HCoV-NL63 spike protein was tested using the same method.

### 2.9. Cytotoxicity Assay

The cytotoxicity of AuNP-HA-NIC was evaluated in vitro using the C2C12 cell line, a subclone derived from mouse myoblast cells, through the CCK-8 assay. AuNPs and NIC were prepared as controls, while AuNP-HA(20)-NIC and AuNP-HA(20)-NIC-Apt served as the test samples. Each of these samples was diluted to concentrations of 5, 50, and 500 μg/mL in the cell culture medium. Cells were cultivated in DMEM enriched with 10% FBS and 1% penicillin/streptomycin and incubated at 37 °C in a humidified environment containing 5% CO_2_. The cells were dispensed into 96-well culture plates at a density of 5.0 × 10^3^ cells per well and were cultured for 24 h. After cell attachment, they were treated with either the target samples or controls for another 24 h. Following this, a mixture of CCK-8 reagent and DMEM medium (in a 1:5 ratio) was added to the cells, which were then incubated for an additional 4 h. The absorbance was gauged using a microplate reader (Synergy H1, BioTek, Winooski, VT, USA) at a wavelength of 450 nm. Cell viability was determined using the following equation:

$$Cell\;viability\;\left(\%\right)=\frac{A_{sample}-A_{blank}}{A_{control}-A_{blank}}\times 100$$

Here, *A_sample_* denotes the absorbance of the samples, *A_blank_* represents the absorbance of the blank (comprising DMEM medium and CCK-8 solution), and *A_control_* refers to the absorbance of the control group (non-treated). The experimental procedures were replicated six times, and the reported data embody the mean value along with the standard deviation.

## 3. Results and Discussion

### 3.1. Characterization of AuNP-HA-NIC

**Figure 1:**
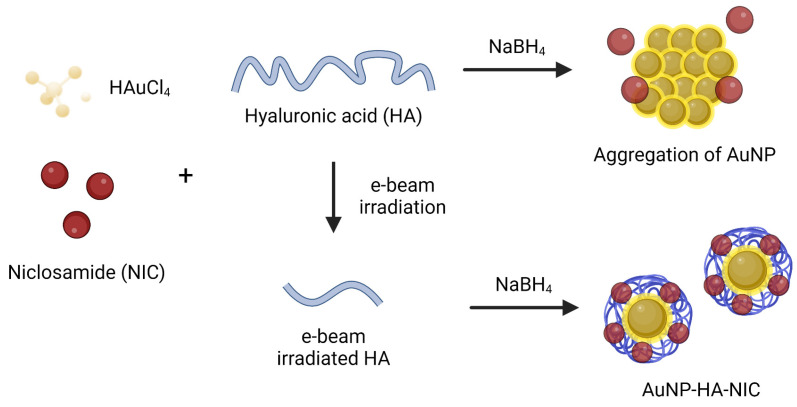
Schematic illustration of the synthesis process for AuNP-HA-NIC.

The overall process of synthesizing the AuNP-HA-NIC is illustrated in Figure 1, and the detailed chemical structures of the NIC, aptamer and HA are depicted in Figure S1. HA of varying molecular weights was prepared by e-beam irradiation at doses of 2, 5, 10, 20, and 50 kGy [26], as shown in Table S2, which presents the molecular weights and viscosities of each irradiated HA solution. Although NIC is insoluble in water but highly soluble in organic solvents such as ethanol, we initially confirmed that gold nanoparticles could be synthesized in ethanol-based solvents. However, redox reactions do not occur well in pure organic solvents, making it difficult to synthesize nanoparticles. For this reason, we found the optimal conditions and confirmed that nanoparticles are well synthesized in a 97% ethanol solvent without NIC precipitation (Figure S2). In this optimized method, HA of varying molecular weights followed by the reduction of HAuCl_4_ in DW with NaBH_4_ were used to form AuNPs. Niclosamide was then incorporated into the AuNPs in an ethanol (EtOH) solution. The final product consisted of AuNP-HA with incorporated niclosamide and an attached targeting aptamer, designed for selective targeting of the SARS-CoV-2 spike protein.

**Figure 2:**
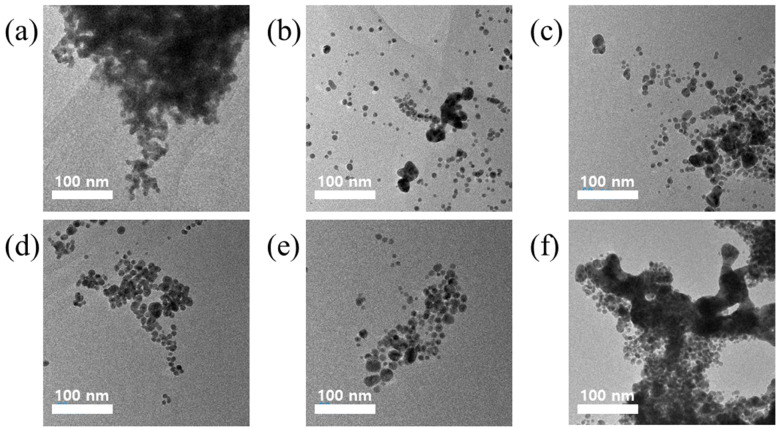
TEM images of AuNP-HA-NIC synthesized with varying e-beam irradiation doses: (**a**) AuNP-HA(0)-NIC, (**b**) AuNP-HA(2 kGy)-NIC, (**c**) AuNP-HA(5 kGy)-NIC, (**d**) AuNP-HA(10 kGy)-NIC, (**e**) AuNP-HA(20 kGy)-NIC, and (**f**) AuNP-HA(50 kGy)-NIC.

As shown in Figure 2, the physical morphology of the NIC-incorporated HA-coated AuNPs (AuNP-HA-NIC) was investigated. The samples were labeled as AuNP-HA(X)-NIC, where X represents the e-beam irradiation dose with a kGy unit. Spherical shapes and smooth surfaces were observed for the particles of the AuNP-HA(2, 5, 10, and 20)-NIC, with sizes ranging from approximately 5 to 20 nm. Conversely, the AuNP-HA(0 and 50)-NIC particles exhibited severe aggregation of numerous smaller particles. As illustrated in Figure S3, the NIC particles are colored yellow, and the AuNP-HA(0)-NIC also exhibits a yellow color, indicating that the particles are poorly formed, similar to the TEM image. In addition, the AuNP-HA(5, 10, and 20)-NIC in DW after ethanol washing showed a clear red color without precipitation, indicating that the particles were evenly dispersed.

The size of drug delivery carriers is a paramount factor in crafting antiviral medications, given that the engineered particles must infiltrate virally infected cells without negatively impacting surrounding healthy cells [30]. To attain a clear understanding of size distribution, dynamic light scattering (DLS) analysis was performed on all AuNP-HA-NIC formulations [26]. The particle-size ranges for AuNP-HA(5, 10, 20)-NIC were 7.9–16.9 nm (average size: 14.5 nm), 6.7–25.4 nm (average size: 14.7 nm), and 5.9–24.3 nm (average size: 14.6 nm), respectively. In contrast, the hydrodynamic size of AuNP-HA(2)-NIC was significantly larger, at 308.1 nm. The AuNP-HA(0, 50)-NIC formulations showcased heavy aggregation, aligning with the conclusions derived from the TEM images. These formulations failed to adequately incorporate the NIC, resulting in inadequate particle formation. Smaller particle sizes, approximately 15 nm, are highly favorable for AuNP-HA-NIC, as they enable seamless entry into infected cells, promoting antiviral actions [31].

**Figure 3:**
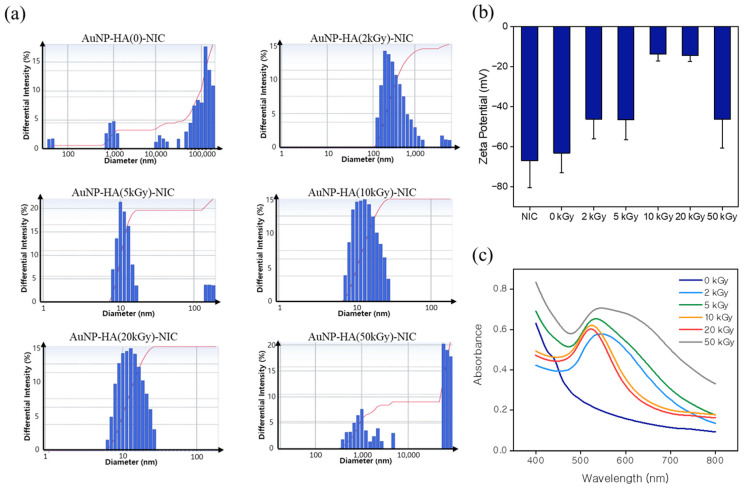
Characterization of AuNP-HA-NIC synthesized with varying e-beam irradiation doses. (**a**) Differential light scattering (DLS) size distribution of AuNP-HA-NIC: AuNP-HA(0), AuNP-HA(2 kGy), AuNP-HA(5 kGy), AuNP-HA(10 kGy), AuNP-HA(20 kGy), and AuNP-HA(50 kGy). (**b**) Zeta potential measurements of AuNP-HA-NIC at different e-beam irradiation doses, indicating the surface charge of the particles. (**c**) UV–Vis absorption spectra of AuNP-HA-NIC, illustrating the effect of e-beam irradiation dose on the optical properties of the nanoparticles.

Figure 3b demonstrates that the zeta potential of the AuNP-HA-NIC possesses negative potential at pH 7.0, confirming that the HA particles were fully coated on the AuNP surface. The surface charge is critical in the interaction between nanoparticles and biological substances. The negative surface of AuNP-HA-NIC is likely to enhance the stability of the nanoparticles in systemic circulation [32]. Furthermore, AuNP-HA(0, 2, 5, and 50)-NIC exhibited a significantly more negative surface charge compared to AuNP-HA(10 and 20). This disparity in surface charge could be due to the presence of NIC, which inherently has a high negative charge. Figure 3c displays the UV–Vis spectra of the nanoparticles. AuNP-HA(5, 10, and 20)-NIC demonstrate a characteristic absorption band within the 500–600 nm range, with a distinct narrow half-peak width. Conversely, AuNP-HA(2 and 50)-NIC exhibit red-shifted absorption, signifying particle aggregation. In the case of AuNP-HA(0)-NIC, the absorption band was absent, implying that nanoparticles were not formed.

### 3.2. Drug Encapsulation Efficiency and Drug Loading Capacity

**Figure 4:**
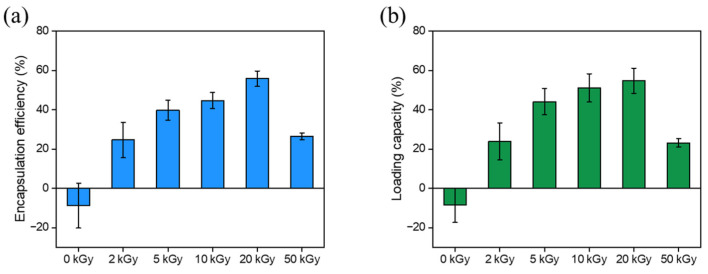
Encapsulation efficiency and loading capacity of AuNP-HA-NIC synthesized with varying e-beam irradiation doses. (**a**) Encapsulation efficiency (%) and (**b**) capacity (%) of AuNP-HA-NIC at 0, 2, 5, 10, 20, and 50 kGy doses.

The drug encapsulation efficiency (DEE) and drug loading capacity (DLC) of AuNP-HA-NIC were evaluated by measuring the weights of the initial input amount of NIC, free NIC in the supernatants, and the synthesized nanoparticles. DEE reflects the proportion of drugs integrated into the synthesized nanoparticles relative to the initial amount of drug input, while DLC denotes the mass ratio of drugs to the synthesized nanoparticles. As shown in Figure 4, the DEEs and DLCs exhibited very similar behavior. For AuNP-HA(20)-NIC, more than half of the NIC was successfully incorporated into the particles. In the case of AuNP-HA(0)-NIC, it was confirmed that no NIC was loaded.

With low-molecular-weight HA, as many covalent bonds constituting the polymer are decomposed compared to high-molecular-weight HA, the contribution of intermolecular interactions surpasses that of covalent bonds when synthesizing the HA layer of the AuNP-HA. Consequently, NIC might be more easily incorporated into the HA layer composed of low-molecular-weight HA, resulting in an increase in both DEE and DLC. On the other hand, AuNP-HA(50)-NIC exhibited low DEE and DLC due to a decrease in the specific surface area of the particles caused by aggregation, resulting in insufficient drug entrapment.

### 3.3. In Vitro Drug Release Study

**Figure 5:**
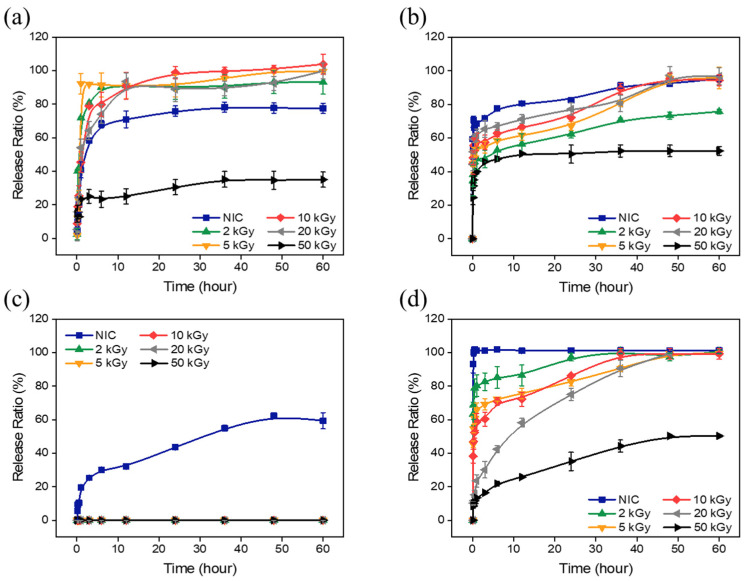
In vitro release profiles of niclosamide from AuNP-HA-NIC synthesized with varying e-beam irradiation doses under different conditions. (**a**) Release in PBS (pH 7.4) containing 0.5% tween 20, (**b**) release in simulated plasma solution (pH 7.4), (**c**) release in simulated gastric fluid (pH 1.5), and (**d**) release in simulated intestinal fluid (pH 6.8).

To verify NIC release characteristics of the AuNP-HA-NICs, an in vitro NIC release study was conducted for both intravenous and oral administration cases. For intravenous administration, a solution mimicking blood was used, while for oral administration, solutions simulating gastric and intestinal fluids were prepared. Figure 5a shows the time-dependent release profiles of NIC in general bio-fluid, PBS (pH 7.4), containing 0.5% (*wt*/*wt*) tween 20. Except AuNP-HA(50)-NIC, the AuNP-HA-NICs released over 60% of NIC in PBS within 1 h, with no additional release observed after 30 h. In contrast, the release rate for AuNP-HA(50)-NIC was notably low, below 30%. For AuNP-HA(0)-NIC, since the NIC was not encapsulated in the nanoparticles, it was excluded from the NIC release test conditions. Furthermore, it was found that the release of NIC from AuNP-HA(2, 5, 10, and 20)-NIC was enhanced as the e-beam irradiation dose of HA increased.

To predict the drug release behavior for intravenous administration, the AuNP-HA-NICs were treated with the simulated plasma solution (Figure 5b). For AuNP-HA(5, 10, and 20)-NIC, there was a rapid release in the first 2 h, followed by a gradual release of the remaining NIC, resulting in a high release rate of more than 90% at 50 h. However, AuNP-HA(2 and 50)-NIC had lower maximum release rates, 70% and 50%, respectively, unlike AuNP-HA(5, 10, and 20)-NIC.

For oral administration, simulated gastric and simulated intestinal fluids were used to predict in vivo drug release behavior (Figure 5c,d). Negligible release was observed in the simulated gastric fluid, whereas leaching of NIC was observed for free NIC. In the simulated intestinal fluid, unlike the simulated gastric fluid, we found that the release was efficient. The initial release behavior varied depending on the molecular weight of HA, with AuNP-HA(50)-NIC showing a significantly lower release of NIC. For AuNP-HA(2, 5, 10, and 20)-NIC, the smaller the molecular weight of HA, the slower the release, and finally, more than 95% of the NIC was released.

These results confirm that the AuNP-HA drug carriers can be properly transported to the intestine without loss of drugs through the digestive system by oral administration, and that the percentage of drug release can be easily varied depending on the e-beam irradiation dose of HA. The drug release rate of the AuNP-HA drug carriers changes with pH; as the pH increases, the HA matrix begins to swell due to the dissolution of carboxyl groups (COO-) in the HA, enabling the release of NIC from AuNP-HA. Oral intake of NIC is influenced by the physiological environment. The pH gradient changes across the digestive tract from the stomach to the intestine [33,34]. In the acidic gastric environment, several pH-induced reactions, including oxidation, hydrolysis, and deamination, can reduce drug efficacy. However, in the case of AuNP-HA-NIC, HA inhibits drug release under acidic conditions in the stomach and promotes successful release at high pH in the intestine, minimizing systemic adverse effects and increasing therapeutic efficacy.

### 3.4. Targeted Drug Delivery

**Figure 6:**
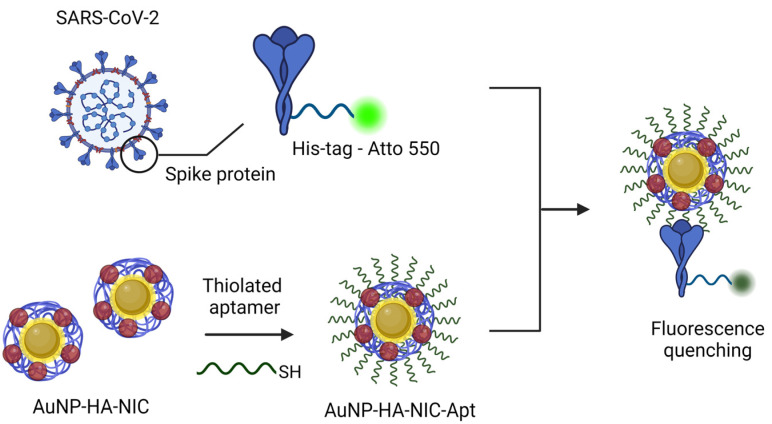
Schematic representation of the targeting mechanism of AuNP-HA-NIC-Apt.

As NIC is a drug that act directly on the virus, targeted delivery to SARS-CoV-2 is necessary to improve the therapeutic effectiveness of COVID-19. To achieve targeted delivery of NIC, an aptamer was applied to AuNP-HA(20)-NIC that can selectively target the spike protein of SARS-CoV-2. As depicted in Figure 6, the His-tagged SARS-CoV-2 spike protein was conjugated with Ni-NTA-Atto 550 to perform a fluorescence quenching assay demonstrating the specific binding of AuNP-HA-NIC-Apt to the SARS-CoV-2 spike protein. This assay shows the interaction with the targeting aptamer, resulting in fluorescence quenching due to specific binding. The phenomenon analyzed is that when AuNP-HA-NIC-Apt and the spike protein bind, the fluorescence is quenched by the AuNPs, whereas when they cannot bind, the fluorescence intensity does not change.

**Figure 7:**
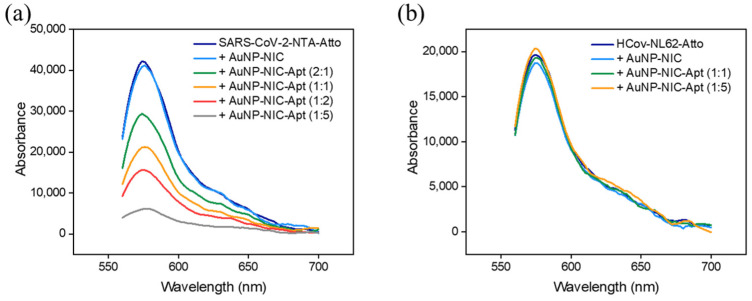
Fluorescence quenching of NTA-Atto 550-labeled spike proteins upon binding to AuNP-HA-NIC-Apt. Fluorescence intensity observed at different target-to-particle ratios for (**a**) SARS-CoV-2 spike protein and (**b**) HCoV-NL63 spike protein.

As shown in Figure 7a, in the AuNP-HA-NIC without the targeting aptamer, the fluorescence of the spike protein remained unchanged, similar to the control, while the fluorescence signals were quenched by using AuNP-HA-NIC-Apt. By varying the target–particle ratios of 2:1, 1:1, 1:2, and 1:5, it was observed that higher quantities led to more fluorescence quenching due to binding with the spike protein. This result confirmed that AuNP-HA-NIC-Apt effectively targets the SARS-CoV-2 spike protein. Conversely, in the case of the non-targeted human coronavirus spike protein, HCoV-NL63, no fluorescence quenching was observed even when AuNP-HA-NIC-Apt was introduced (Figure 7b). These results clearly confirm that AuNP-HA-NIC-Apt exhibits specific binding to the SARS-CoV-2 spike protein.

### 3.5. Cytotoxicity Test

**Figure 8:**
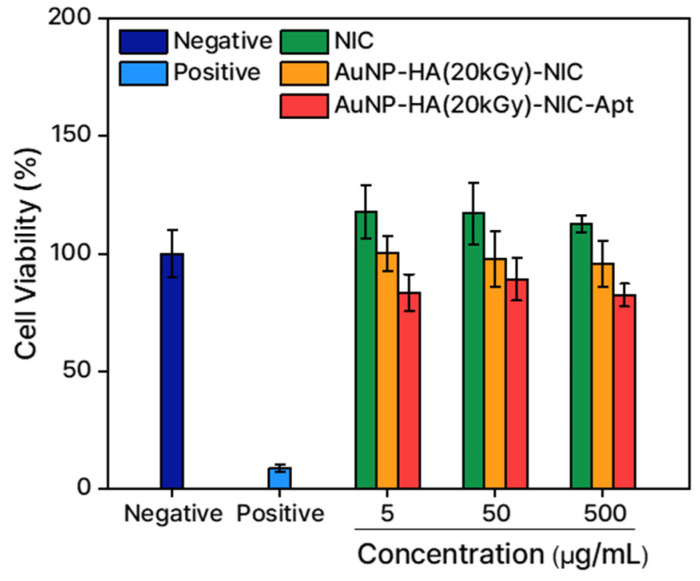
In vitro cell viability assay of AuNP-HA(20)-NIC and AuNP-HA(20)-NIC-Apt at different concentrations (5, 50, and 500 μg/mL) with negative control (untreated cells) and positive control (NiSO_4_-treated cells).

An in vitro cytotoxicity test was performed to assess the toxicity of the targeted drug delivery nanocarrier. In this assay, a non-treated condition and NiSO_4_-treated condition were employed as a negative control and a positive control, respectively. For the samples, AuNP-HA(20)-NIC and AuNP-HA(20)-NIC-Apt were administered to each well at concentrations of 5, 50, and 500 μg/mL for 24 h. Figure 8 shows the cell viability results of free NIC and the samples in comparison to the assay control. The reliability of this assay was sufficiently secured because the negative control confirmed that the cells grew well, while the positive control confirmed that the cell viability was less than 10%.

For the samples, it was found that all had the same level of cell viability. Although there were slight decreases in cell viability as the amount of NIC increased, it was clear that this change was due to the pharmacological action and not the toxicity of the drug delivery system itself. As a result, we demonstrated that AuNP-HA-based drug delivery vehicles can be used as effective carriers to deliver poorly water-soluble drugs without exhibiting any significant toxicity.

## 4. Conclusions

In this study, we developed a AuNP-HA-NIC system as a novel drug delivery platform aimed at combating the SARS-CoV-2 virus. By employing a one-pot synthesis strategy, we successfully loaded NIC onto AuNP-HA, enhancing bioavailability and achieving controlled release. The use of various molecular weights of HA obtained through e-beam irradiation resulted in the most stable nanoparticles, particularly at an irradiation level of 20 kGy. The AuNP-HA-NIC system demonstrated high DEE and DLC, with its effectiveness further validated by physicochemical analysis. The system’s pH responsiveness ensures targeted drug release in the intestinal tract, minimizing premature release in the stomach. In addition, the targeted drug delivery was achieved by attaching an aptamer to the AuNPs, which specifically binds to the SARS-CoV-2 spike protein, ensuring precise targeting and improved therapeutic efficacy. The low cytotoxicity of the nanoparticle system, as evidenced by cell viability tests, underscores its potential as a safe and effective antiviral agent. As further in vivo testing and optimization continue, the AuNP-HA-NIC system holds significant promise for advancing antiviral therapeutics, particularly in the fight against COVID-19.

## Data Availability

The datasets generated and/or analyzed during the current study are available from the corresponding authors on reasonable request.

## Supplementary Materials

The following supporting information can be downloaded at https://www.mdpi.com/article/10.3390/pharmaceutics16101288/s1, Figure S1: (a) Chemical structure of niclosamide, (b) Sequence and secondary structure of SARS-CoV-2 spike protein targeting aptamer, and (c) Chemical structure of hyaluronic acid and its degradation profile after e-beam irradiation; Figure S2: Solubility and dispersion stability of AuNP-HA-NIC nanoparticles synthesized in different ethanol concentrations (100%, 97.09%, 96.15%, 95.24%, 92.60%, and 91.74%); Figure S3: Images demonstrating the dispersion and color change of NIC-incorporated HA-coated AuNPs (AuNP-HA-NIC) synthesized with different e-beam irradiation doses (0, 2, 5, 10, 20, and 50 kGy) in 97% EtOH and DW; Table S1: Aptamers sequence used in this work; Table S2: Characterization results of e-beam-irradiated HAs. (Molecular weights were determined by MALS system and viscosity (10 mg/mL, 22.5 °C)).
