# Supplementary material for: Development of Targeted Drug Delivery System for the Treatment of SARS-CoV-2 Using Aptamer-Conjugated Gold Nanoparticles
Source: Pharmaceutics. 2024 Sep 30;16(10):1288. doi: 10.3390/pharmaceutics16101288 (PMC11510760; doi:10.3390/pharmaceutics16101288)
Supplement: Supplementary file 1 [file pharmaceutics-16-01288-s001.zip › pharmaceutics-3200594-supplementary.pdf]

Supplementary Materials: Development of Targeted Drug Delivery System for the Treatment of SARS-CoV-2 Using Aptamer-Conjugated Gold Nanoparticles

Junghun Park [1,†], Hyogu Han [2,3,†] and Jun Ki Ahn [2,*]

Table S1. Aptamers sequence used in this work.

**Aptamer sequence (5′ → 3′)**
ATCCAGAGTGACGCAGCACCGACCTTGTGCTTTGGGAG-TGCTGGTCCAAGGGCGTTAATGGACACGGTGGCTTAGT

Table S2. Characterization results of e-beam-irradiated HAs. (Molecular weights were determined by MALS system and viscosity (10 mg/mL, 22.5 °C)).

e-beam Dose (kGy)	Molecular weight (Da)	Viscosity (cP)
**0**	$9.761\times10^5 \pm 10.1\%$	5140
**2**	$5.544\times10^4 \pm 5.7\%$	333
**5**	$4.078\times10^4 \pm 5.1\%$	114
**10**	$2.111\times10^4 \pm 9.0\%$	95
**20**	$1.174\times10^4 \pm 8.0\%$	19
**50**	$3.927\times10^3 \pm 15.9\%$	5

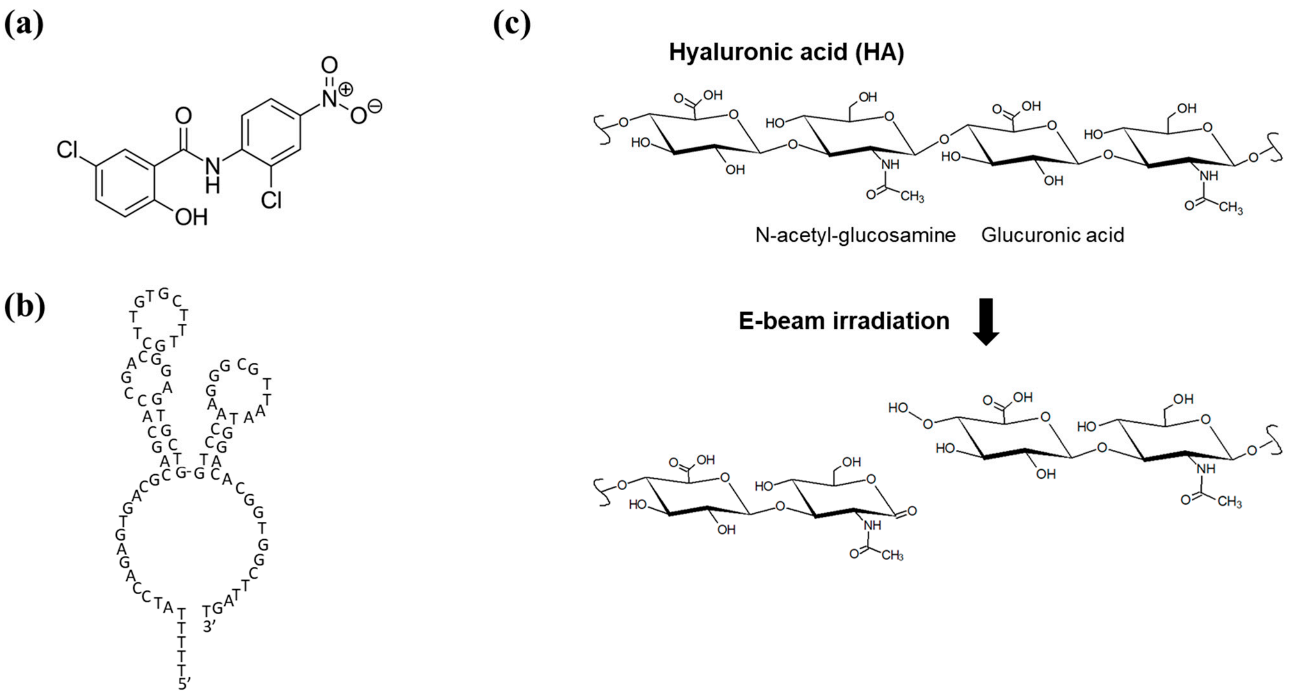

Figure S1. (a) Chemical structure of niclosamide, (b) Sequence and secondary structure of SARS-CoV-2 spike protein targeting aptamer, and (c) Chemical structure of hyaluronic acid and its degradation profile after e-beam irradiation.

<table>
<tr><th rowspan="2">EtOH Solvent Ratio</th><th colspan="3">NIC Concentration (mg/mL)</th><th rowspan="2">EtOH Solvent Ratio</th><th colspan="3">NIC Concentration (mg/mL)</th></tr>
<tr><th>10</th><th>8</th><th>5</th><th>10</th><th>8</th><th>5</th></tr>
<tr><td>100%</td><td colspan="3">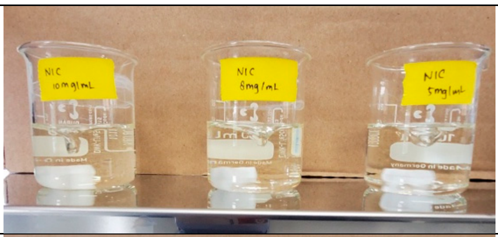</td><td>95.24%</td><td colspan="3">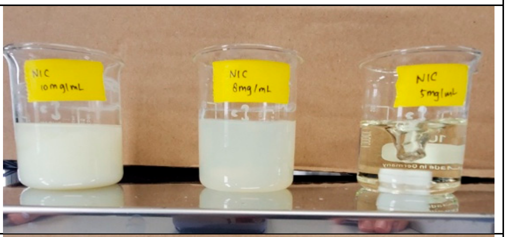</td></tr>
<tr><td>97.09%</td><td colspan="3">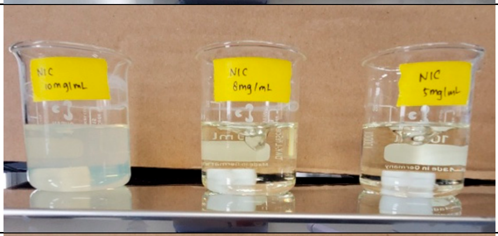</td><td>92.60%</td><td colspan="3">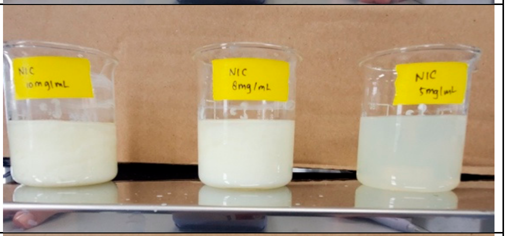</td></tr>
<tr><td>96.15%</td><td colspan="3">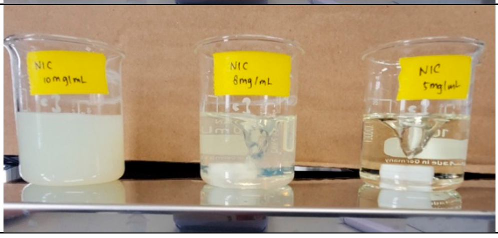</td><td>91.74%</td><td colspan="3">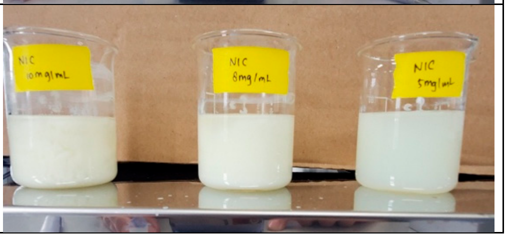</td></tr>
</table>

Figure S2. Solubility and dispersion stability of AuNP-HA-NIC nanoparticles synthesized in different ethanol concentrations (100%, 97.09%, 96.15%, 95.24%, 92.60%, and 91.74%). The images illustrate the optimal conditions for synthesizing nanoparticles in 97% ethanol solvent without NIC precipitation, confirming stable and well-dispersed nanoparticles.

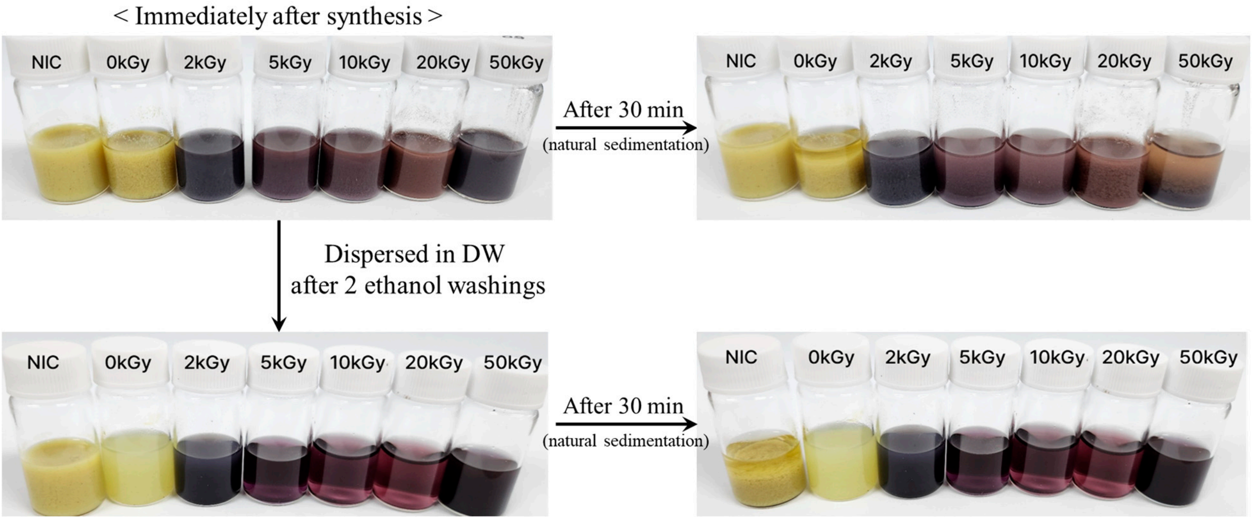

Figure S3. Images demonstrating the dispersion and color change of NIC-incorporated HA-coated AuNPs (AuNP-HA-NIC) synthesized with different e-beam irradiation doses (0, 2, 5, 10, 20, and 50 kGy) in 97% EtOH and DW.
